# Compost Dairy Barn Layout and Management Recommendations in Kentucky: A Descriptive Study

**DOI:** 10.3390/ani12233324

**Published:** 2022-11-28

**Authors:** Flávio Alves Damasceno, George B. Day, Joseph L. Taraba, Carlos Eduardo Alves Oliveira, Rafaella Resende Andrade, Karen Dal Magro Frigeri, Frederico Márcio Corrêa Vieira, Matteo Barbari, Gianluca Bambi

**Affiliations:** 1 Department of Engineering, Federal University of Lavras (UFLA), Lavras 37200-900, MG, Brazil; 2 Department of Agricultural Engineering, University of Kentucky, Lexington, KY 40502, USA; 3 Department of Agricultural Engineering, Federal University of Viçosa (UFV), Viçosa 36570-900, MG, Brazil; 4 Department of Agriculture, Food, Environment and Forestry, University of Firenze, 50145 Firenze, Italy; 5 Biometeorology Study Group, Federal University of Technology—Paraná (UTFPR), Dois Vizinhos 85660-000, PR, Brazil

**Keywords:** dairy cow, bedding, compost barn, structure, design

## Abstract

**Simple Summary:**

Here, we aimed to characterize the structural features, to descript the bedding material, and to observe the management practices of compost (CBP) barns in the state of Kentucky (USA). These systems allow thermal comfort for animals, as well as better hygiene conditions for cows. All CBP barns showed structural variations. Sawdust and wood shavings were the most used materials in beds and coliforms *E. coli*, *Bacillus*, and *Streptococcus* were present in the CBP barns with a lower moisture content. In addition, the most frequently cited benefits of the CBP barn include increased cow comfort compared to free stalls; increased cow cleanliness; low maintenance; its ability to work well for heifers and lame, fresh, problem, and old cows; and its ability to allow natural resting positions with no free stalls.

**Abstract:**

This study was conducted to describe the building layout and dimensions, characterize the bedding material, and observe the management practices in 42 compost-bedded pack (CBP) barns in Kentucky (USA). The average herd size found in the study was 90 cows and the breeds consisted of Jersey (6.8%), Holstein (72.7%), and mixed (20.5%). The average CBP barn dimensions were 49.1 m (length) by 21.9 m (width). Many of these barns had feed alleys and driveways; overshot ridges with frequent orientation from NE to SW; and green sawdust, kiln-dried sawdust, or a mixture of both as the most common bedding materials. The bed-turning process was performed mechanically at depths of less than 0.25 m, and the loading of fresh material was performed every one to five weeks, varying by season, weather conditions, barn size, and cow density. The average bedding moisture content was found to be 59.0% (wet bulb—w.b.) and ranged from 36.2 to 71.8%. Coliforms were not present in barns that had a higher compost temperature, and the *E. coli*, *Bacillus*, and *Streptococcus* counts were higher in the barns that had a lower moisture content. In conclusion, it was observed that heterogeneous management was used among the barns and that the producers were satisfied with the compost barn system.

## 1. Introduction

In the late 1980s, innovative dairy producers in the state of Virginia introduced a new variation on the loose-housing dairy system, generally referred to as a compost-bedded pack (CBP) barn. Since then, CBP barns have been used in many states in the United States, especially in the Midwest and Northeast, and in other countries such as Japan, China, Germany, Italy, the Netherlands, Israel [1], and recently, in Brazil [2,3,4]. This significant increase is indicative that a CBP barn may be a reasonable, economically feasible alternative type of dairy housing facility for dairy producers wanting to upgrade or modernize their milking herd facilities. As a consequence, factors based on dairy producer experiences such as the current design, the management recommendations for manure compost packs, animal behavior, herd health, and milk production has been described in some publications [5,6]. Additional field observations have also been reported in several countries [7,8,9,10,11].

The adoption of the CBP system has increased significantly in recent years, and the main reasons that have led so many producers to opt for this type of confinement system are: thermal comfort and well-being, increased productive and reproductive efficiency of the herd, improvements to milk quality, reduced hoof problems, fewer problems with ectoparasites and insects, a better initial construction cost, and better manure management. On the other hand, depending on the country, the CBP system has some disadvantages, such as: difficulty finding bedding quantity and quality, as well as rising costs of bedding acquisition; the need for a tractor and agricultural implements for bed management, with a consequential increase in maintenance costs; a greater demand for electricity, due to the need for a greater number of fans, thus increasing fixed costs; and the need to exploit animals with a high productive potential to dilute the costs of the implementation and maintenance of the system [12].

Compost packs produced by composting are stirred three times daily to incorporate the manure, provide a fresh, soft surface, and aerate the pack to encourage aerobic microbial activity in the pack [9]. Compared to windrow composting, the CBP system has a larger surface area to heat-generating volume, and thus results in a greater heat loss. Since the temperature is one of the most important CBP operational parameters, understanding the thermal properties and heat balance in compost systems is required to design and properly control the composting processes [9,13]. At the same time, the key to keeping a CBP barn in good working order includes a properly designed structure, improved ventilation, frequent bedding turning, optimal humidity in the bedding package, proper biodegradability of the bedding material with animal waste, and optimal stocking density [1]. 

Therefore, the effectiveness of the composting process is dependent upon the environmental conditions, including the oxygen content, the moisture, the temperature, the amount of organic matter, and the size and activity of microbial populations present within the CBP barn [14]. Composting increases the bedding temperature, which reduces pathogenic microbial populations and decreases bedding moisture by increasing the drying rate [1,15]. The large numbers of microorganisms and high microbial activity in the compost bedding are largely a reflection of manure deposition, bedding chemical properties (available carbon and nitrogen), and water content [14]. Facultative anaerobic bacteria also are more numerous on the surface of CBP material and constitute a larger proportion of the total microbial population in tilled or stirred compost [16].

Despite these advances in research, we found a lack of knowledge about the air quality inside facilities for dairy cows, especially for the CBP system. Further research is needed to assess and correlate detailed information about the building structure, bedding management, and composting parameters of these housing systems. Additionally, it is necessary to determine the most significant factors for achieving CBP success.

We aimed to conduct a study of 42 compost-bedded pack barns in Kentucky (USA) to describe the building layout and dimensions, characterize the bedding material, and determine the major interactive factors in the success of bedding composting. We hypothesized that compost barns have experienced a great acceptance among farmers and that the environmental conditions in a well-managed system result in a lower incidence of coliforms and more comfortable and cleaner cows.

## 2. Materials and Methods

### 2.1. Characterization and Description of CBP Barns

#### 2.1.1. Barn Facility Measurements

**Figure 1:**
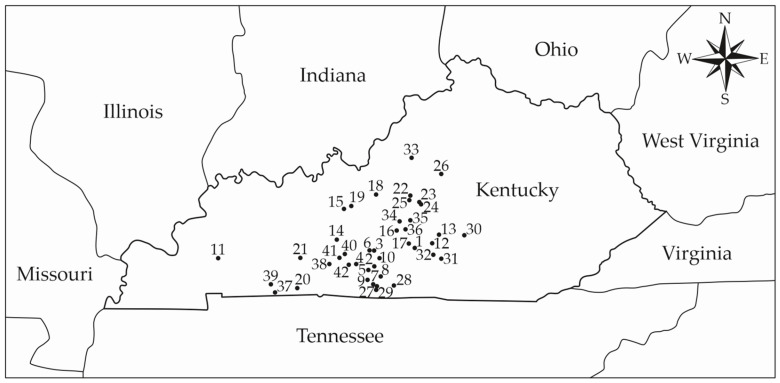
Distribution of the assessed CBP barns in the state of Kentucky (USA). 1 to 42—number of the assessed CBP barn.

The database used in this study comprised data collected from different CBP barns distributed throughout the state of Kentucky—U.S. (Figure 1). Initially, contact was made with the producers and the project was presented. Once accepted, the team was directed to the farm for data collection. In this study, if a farmer described the barn as a compost-bedded pack barn but the pack was rarely or never stirred, the barn was not included in any data analyses. Thus, forty-two farms participated in the study.

The barn dimensions were measured using a steel tape measure, surveyor’s tape, a level rod, and a measuring wheel. The roof pitch was determined using a level and a framing square placed directly on the roof. Two fiberglass ladders were used to allow access to the roof structures and ventilation fans. Digital photography was used extensively to document the interior and exterior structures and systems to assist in the accurate development of a database of the barn structure, layout, and ventilation details. All information was recorded in a notebook.

#### 2.1.2. Environmental Measurements

**Figure 2:**
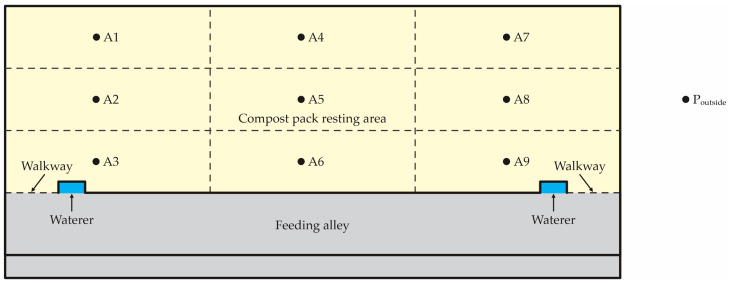
Nine grid spaces (A1–A9) and one outside point (P_outside_) of environmental measurements and sample collections inside of the barns.

The air temperature, relative humidity (RH), air velocity, and wind direction were measured inside and outside of each barn. At nine locations within a barn, measurements were taken in the compost pack area at two different heights (0.05 and 1.2 m) one time on the day of the site visit. The air temperature and RH were measured using a weather meter (accuracy of ±1 °C; Kestrel^®^, model 4000, Sylvan Lake, MI, USA). The air velocity was measured using a hot wire anemometer (accuracy of ±0.01 m∙s^−1^; Testo^®^, model 425, Sparta, NJ, USA). The air direction was measured with a weathervane. Sampling points (A1 to A9) represent the approximate center of nine grid spaces within the manure pack. The grids were established based on post spacing along with the pack and were generally along the center of the pack (Figure 2). Data from the external environment were collected at a representative location close to the CBP barn and adapted to environmental conditions.

#### 2.1.3. Bedding Temperature Measurements

The bedding temperature was measured at nine locations as previously described (Figure 2) across each pack at the surface and at two different depths (0.10 and 0.20 m). The bedding surface temperature was measured using two types of sensors. An infrared thermometer (accuracy of ±1 °C; Fluke^®^, model 62, Everett, WA, USA) and a compact thermal camera (accuracy of ±0.1 °C; Extech^®^, Flir i5, Waltham, MA, USA) were used. The bedding temperature was measured using a thermocouple-based thermometer (0.22 m length and an accuracy of ±2.2 °C; Fluke Inc., model 87, Everett, WA, USA).

### 2.2. Bedding Moisture Analysis

Samples of pack bedding were collected from a mixture of compost on the top (0.10 m) in nine different locations inside the barns using plastic bags (Ziploc^®^, Double Zipper, Racine, WI, USA). Data collection was carried out in the winter of 2010, during which period the management of the litter in the CBP system is the most difficult. Therefore, the worst condition to evaluate the CBP barns was chosen. Samples were collected using an iron hoe with a wooden handle and a soil auger before stirring the bed, so as not to influence all the moisture and bacteria present. The bedding samples were cooled on ice upon collection and refrigerated when returned to the lab at 1.0 °C. 

The bedding compost moisture content throughout this study was measured by drying at 105 °C for approximately 24 h. The moisture content (dry basis) of bedding compost was defined by Equation (1) [17]:

(1) $$MC_{dry-base}\left(\%\right)=\frac{m_{w}-m_{d}}{m_{d}}\times 100$$

where $m_{w}$ is the mass of the wet material (in g) and $m_{d}$ is the mass of the dry material (in g), determined by the placement of the sample in a convective oven at 105 °C for 24 h. $MC_{dry-base}$ is expressed as a percentage.

### 2.3. Bedding Material Bacterial Count Analysis

Bedding material samples were collected during a single site visit from nine evenly distributed locations throughout each CBP barn (Figure 1). The researchers collected 118.3 cm^3^ of surface layer bedding material from each location (total 1064.7 cm^3^) using a 59.1 cm^3^ measuring cup (Everyday Living™, The Kroger Co., Cincinnati, OH, USA) in a 3.8 L plastic bag (Ziploc^®^, Slider Storage and Freezer Bags with SmartZip^®^ Seal, Racine, WI, USA) and thoroughly mixed the material to create a composite sample representative of the entire CBP. The samples were stored in a −40 °C freezer until at least 20 composite samples were collected and available for analysis. The sample preparation consisted of diluting the material by mixing 25 g of bedding material with 225 g of 0.1% peptone solution to a 1:10 dilution.

The mixture was hand-mixed until the bedding material was well-suspended within the peptone solution. Further serial dilutions were performed to obtain countable plates. To determine the total coliform species and the *Escherichia coli* count, researchers added 1 mL of the appropriate dilution to 3M™ Petrifilm™ *E. coli*/coliform count plates (3M™ Microbiology Products, St. Paul, MN, USA) and incubated the plates at 35 °C for 24 h. Colony-forming units (cfus) were counted manually, obtaining both a coliform and *E. coli* count. The researchers determined the *Streptococcal* species count by spiral plating (Eddy Jet, IUL Instruments, I.L.S., Leerdam, The Netherlands) the diluted material onto a plate containing TKT agar prepared in the lab. The plates were incubated for 48 h at 35 °C. For *Staphylococcal* species, BBL™ Columbia CNA agar (Becton, Dickinson and Company, Franklin Lakes, NJ, USA) was prepared according to the manufacturer’s directions.

The diluted material was spread across the plate surface. The plates were incubated for 48 h at 35 °C and then flooded with peroxide. Catalase-positive colonies were counted as *Staphylococcal* species. *Bacillus* species counts were ascertained by spiral plating (Eddy Jet, IUL Instruments, I.L.S., Leerdam, The Netherlands) the diluted material onto a plate containing Difco™ MYP (mannitol egg yolk polymyxin B) agar (Becton, Dickinson and Company, Franklin Lakes, NJ, USA), prepared according to the manufacturer’s directions. The incubation of the CNA, TKT, and MYP plates occurred at 35 °C for 48 h, with the cfus counted automatically using a colony counter (Flash & Go, IUL Instruments, I.K.S., Leerdam, The Netherlands). All bacterial counts are reported in log_10_ cfu∙g^−1^ on a wet matter basis.

### 2.4. Descriptive Statistics

The MEANS procedure of SAS^®^ (SAS 9.3, SAS Inst. Inc., Cary, NC, USA) was used to calculate the means and standard deviations (SD) of all non-categorical management practices, bacterial counts, ambient and internal barn temperatures and RH, CBP temperatures, and barn designs. All means are reported as the mean ± SD. The FREQ procedure of SAS^®^ was used to calculate the producer comment and management practice frequencies.

## 3. Results

### 3.1. Barn Structure and Layout

The compost dairy barn measurement and layout information collected included: barn and pack dimensions, feed alley dimensions, waterer locations and sizes, fan and light descriptions, stocking density, and sidewall height, as well as other barn specifications.

**Figure 3:**
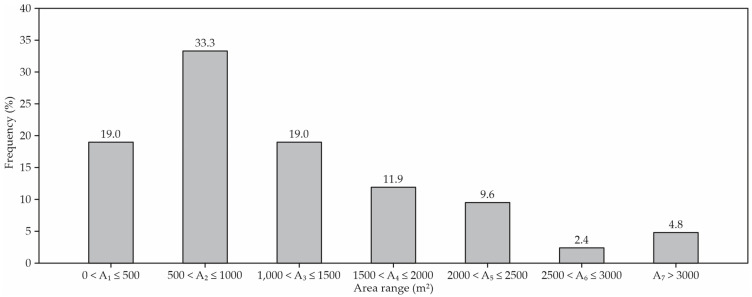
The frequency distribution of compost-bedded pack barn areas.

The average compost bedding pack barn dimensions were 49.1 m (length) by 21.9 m (width). The most frequent barn length was between 25 and 50 m (45.2%) and between 50 and 75 m (33.3%); 11.9% of the barns were higher than 75 m, and 9.5% were less than 15 m. The most frequent barn width was between 15 and 30 m (61.9%), and 19.0% were less than 15 m, 16.7% were from 30 to 45 m, and 2.4% were higher than 75 m. Most compost-bedded pack barns had areas between 500 and 1000 m^2^ (33.3%; Figure 3).

Many of these barns had feed alleys and driveways. Only in 26.2% of the barns, the cows had free access to pasture; 9.5% of the barns were equipped with cow brush. A feed store and anaerobic lagoon were present around these barns in 52.3%. Headlocks were found in 19% of the CBP barns.

In 24% of the compost-bedded pack barns, there was no access to water for the cows. The cows in over 76.0% of the barns were critically short of watering space. In Kentucky, some CBPs were built in old tobacco sheds, and access to water and food for the animals occurred in sheds close to the CBP barn. Regarding the feeder space, 29% of the barns presented dimensions from 0.64 to 0.80 m·cow^−1^. Only 31% of the compost-bedded pack barns had no feed bunks, although most of these had concrete alleyways leading to nearby feed bunks. The available space per animal ranged from 0.2 to 1.5 m∙cow^−1^ in the feed alley, and from 0.04 to 1.55 m∙cow^−1^ in the waterers.

**Figure 4:**
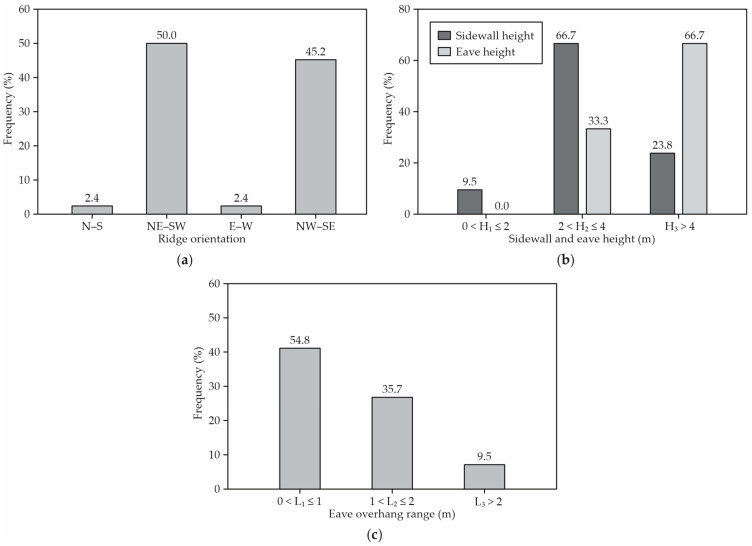
Frequency distribution of (**a**) ridge orientation, (**b**) sidewall and eave height, and (**c**) eave overhang range. N–S: north–south; NE–SW: northeast–southwest; E–W: east–west; and NW–SE: northwest–southeast.

Regarding the ridge orientation, the most frequent orientation was NE–SW (50.0%, Figure 4a), with the next most frequent being NW–SE (45.2%, Figure 4a).

Regarding the slope of the roofs, 54.8% of the barns had a slope of 4:12 and 14.3% of the sheds had a slope of 3:12. The most frequent lateral wall opening height between the sheds was between 2.0 and 4.0 m (66.7%), while the height of the eaves in 66.7% of the barns was more than 4.0 m. Some of these barns had low walls on the outside perimeter to contain the compost, which reduce the effectiveness of the measured eave height (Figure 4b).

Many barns (54.8%) had an eave overhang of less than 1.0 m; 35.7% of the barns had an eave overhang between 1.0 m and 2.0 m; and only 9.5% of the barns had an eave overhang with 2.0 m or more of the sidewall height (Figure 4c).

**Figure 5:**
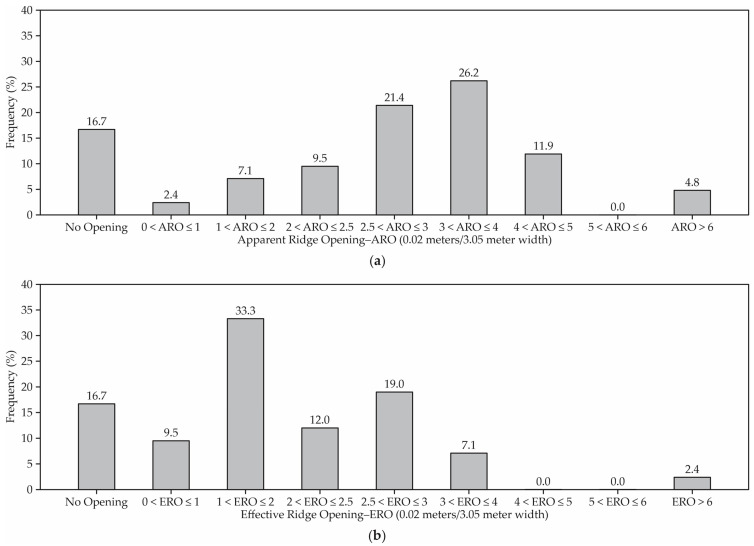
Ridge opening-to-barn width ratio for compost-bedded pack barns: (**a**) apparent ridge opening compost barns—0.02 m/3.05 m width and (**b**) effective ridge opening compost barns—0.02 m/3.05 m width.

The types of ridges found in the study were: overshot ridges (59%), open ridges with a cover (19%), capped ridges (12%), open ridges without a cover (5%), and hoop structures (5%). There are two ridge opening values shown in Figure 5: (**a**) an apparent ridge opening (0.02 m/3.05 m of building width) and (**b**) an effective ridge opening (0.02 m/3.05 m of building width).

Figure 5 indicates the compost barn ridge measurements as a ratio of the ridge opening to barn width (m/3.05 m). A total of 17% of the barns had no ridge opening. Based on the apparent ridge opening (ARO), 18% had an inadequate ridge opening (<2.5 0.02 m/3.05 m), 17% had a marginal ridge opening (2.5 to <0.07 m/3.05 m), and 48% equaled or exceeded the recommended ridge opening [18]. However, based on the effective ridge opening (ERO), 53% had an inadequate ridge opening, 20% had a marginal ridge opening, and only 11% equaled or exceeded the recommended ridge opening.

Most of the compost-bedded pack barns were equipped with box fans (42.8%) for circulation, with 30.9% using natural ventilation, 23.8% using high-volume and low-speed fans (HVLS) for circulation, and 2.5% using tunnel ventilation (hoop structures). Most of the HVLS fans used had a diameter of 7.32 m. The average diameter of the box fans was 0.98 m, ranging from 0.46 m to 1.32 m.

The lighting in the compost-bedded pack barns was noted at each dairy visit. It was found that 41% of the compost-bedded pack barns did not have lights. High-intensity discharge (HID) was the most frequent form of light that producers used. This was followed by fluorescent (14%), compact fluorescent (12%), and incandescent (10%) lighting. The total lighting wattage was also noted. The average power used for lighting in the barns was 124 W, ranging from 13 to 192 W.

### 3.2. Compost-Bedded Pack Management

The highest percentage (45.2%) of barns were bedded with kiln-dried wood shavings or sawdust, followed by green sawdust (42.9%) and a blend of both materials (11.9%). Most of the barns in Kentucky had their compost tilled and aerated to a depth of 0.11 to 0.23 m twice a day in the summer and winter while the cows were at the milking parlor. The most often-used stirring tool was a chisel plough on a small tractor.

On the farms visited, the last addition of bedding varied between one week and six months before the study was carried out. Many farmers cleaned out the pack entirely once a year. In this case, a load of clean material (shavings or sawdust) was added after the removal of the used bedding to a depth of 0.30 m to start the new pack. Typically, a load of fresh material was added every one to five weeks, varying by the season, weather conditions, barn size, and cow density. This data varied due to the supply of material available to each producer. Some farmers left about 0.15 m of old material in the barn to help initiate microbial activity. There was a small number of dairies that added a thin layer of fresh bedding when the bedded pack became moist enough for it to stick to the cows’ bodies after they rose from lying down on the bedded pack.

The average pack dimensions for the 42 compost barns we studied were 47.7 m (±19.1) and 16.1 m (±5.9). The average stocking density in this study was 8.9 m^2^·cow^−1^, ranging from 0.8 to 25.8 m^2^·cow^−1^.

Temperatures of the bedding compost tended to be lower in barns 16, 22, 23, 24, 29, 35, 36, and 38, which utilized green sawdust rather than kiln-dried wood shavings or sawdust for bedding (*t*-test, *p* > 0.05). The temperatures were greater in the areas of the pack that were fluffier and that were not heavily soiled or packed by the cows.

The average bedding moisture content in the top 0.20 m was 59.0% (±9.0), ranging from 36.2 to 71.8%.

### 3.3. Environment Characteristics

**Figure 6:**
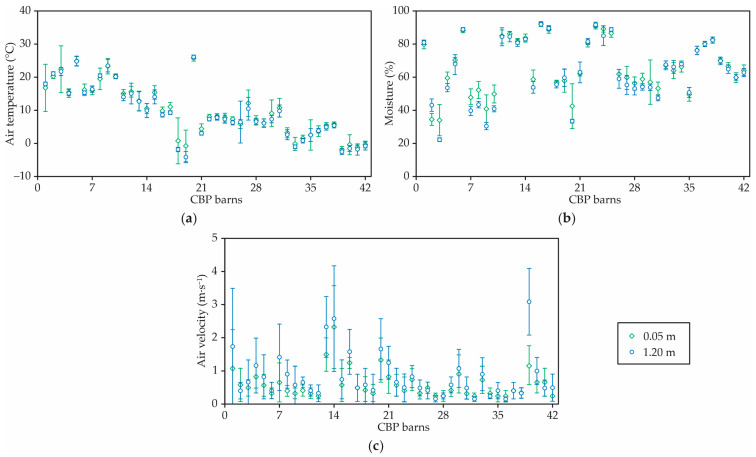
Average of (**a**) air temperatures, (**b**) relative humidity, and (**c**) air velocity measured at two heights (0.05 and 1.20 m) inside 42 CBP barns in Kentucky.

The average temperature gradient (taken at 0.05 m and 1.20 m above the pack) was 0.52 °C∙m^−1^ (±1.0), indicating that there was a reduction in the air temperature along with the height measurement (Figure 6a). A paired *t*-test statistical analysis (*p* < 0.05) indicated that the average air temperatures for the two heights were different. The average surface temperatures on the pack were similar to the average ambient temperature. The air temperatures tended to be lower inside barns that utilized overshot or capped ridges, mechanical ventilation systems, and a ridge direction of SW or NW (Figure 6a).

The average RH gradient in this study was −2.35 %∙m^−1^ (±0.5). Thus, many barns tended to increase in RH along with height. A paired *t*-test statistical analysis indicated that the average RH for the two heights was different (*p* < 0.05). In some barns, sawdust bedding was used in higher amounts to keep the cattle comfortable and dry which increased the barn operating costs (Figure 6b).

The average air velocity gradient was −0.22 m∙s^−1^∙m^−1^ (±0.30), indicating that the air velocity decreased along with the height (Figure 6c). A paired *t*-test statistical analysis (*p* < 0.05) indicated that the average air velocity for the two heights was different.

A considerable difference between the air velocity indoors and outdoors was observed in barns 1, 5, 6, 11, 12, 13, 14, 16, 17, 20, 22, 23, 24, 25, 37, and 38. Buildings and other obstacles (trees, hills, and tractors) around these barns blocked airflow and resulted in a higher RH.

### 3.4. Herd Characteristics

**Figure 7:**
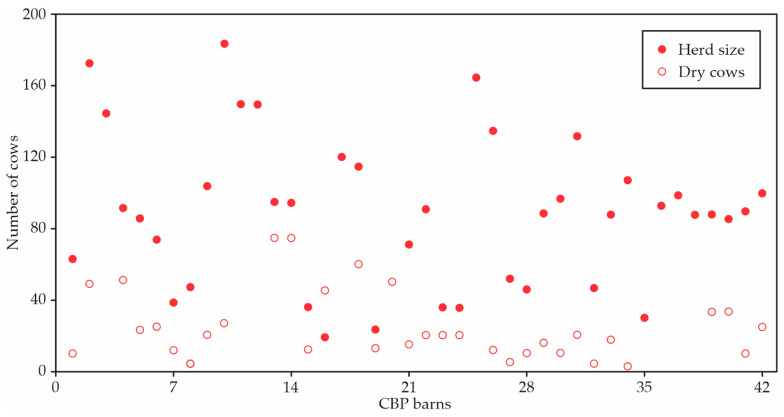
Herd size and number of dry cows in 42 CBP barns.

The cow herd size, as reported by the producers, was adjusted for breed size and milk production. The average herd size on the pack in the study was 89.7 cows, ranging from 19 to 184 cows, and the dry cow numbers ranged from 3 to 75 dry cows, with an average of 25.1 dry cows (Figure 7). Dry cows were housed on pasture, in a separate area or the same area of the compost barn as the lactating cows, or in an alternative housing area. The breeds included Jersey (6.8%), Holstein (72.7%), and mixed (20.5%). The average milk production of the cows was 27.2 kg∙day^−1^ with a mean fat and protein percentage of 3.85% and 2.35%, respectively.

### 3.5. Producer Responses

All the producers that visited were very satisfied with their compost barns. They observed that the cows were more comfortable than in previously used housing systems (24.2%) and that this housing system resulted in increased cow cleanliness (12.1%; Table 1).

However, the results of the survey revealed that producers would change some aspects of the CBP barn. Their main concern was the size or capacity of the barn (25%). Further, many producers indicated the need for retaining walls (10%) and curtains (8.3%; Table 2).

Most producers were concerned about the supply of bedding (Table 3). Many producers were seeking a secure bedding supply (17.7%) and alternative bedding sources that would work in the CBP (9.7%).

The producer recommendations also included the importance of maintaining the pack (8.1%) and stirring the pack two times per day or more frequently (14.5%); using kiln-dried shavings (9.7%); avoiding the use of straw, wheat straw, corn fodder, soybean fodder, or pine as a bedding material (9.7%); and maintaining the pack by keeping the moisture low (8.1%; Table 3). Some producers (4.8%) reported that touring other barns was an important influence on their choice of structure. Producers also reported that after speaking with other producers managing a CBP barn, they created their plans and reviewed free stall plans and designs, economics, and ease of management. Producers learned that using kiln-dried shavings, keeping the pack stirred, and avoiding the use of straw in the bedding would help to prevent other mistakes.

### 3.6. Bacterial Analysis of Composts

**Figure 8:**
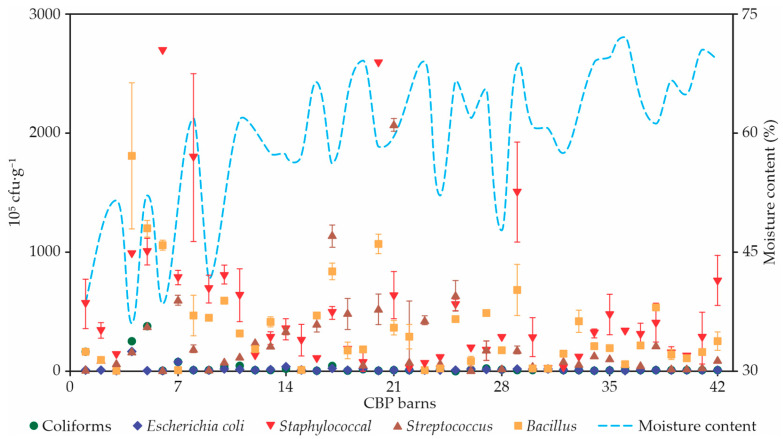
Bacterial analysis of bedding compost samples in 42 CBP barns.

Bedding compost always contained bacteria, but the variation was large between different sampling sites and times (Figure 8). However, coliforms were not present in barn 15, which had a higher compost temperature. The coliform, *E. coli*, *Bacillus*, and *Streptococcus* counts were higher in barn 4, which had a lower moisture content (36.2%). The average coliform, *E. coli*, *Streptococcus*, and *Bacillus* contents in the bedding material of barn 4 were 247.5 ± 12.0 cfu∙g^−1^, 173.0 ± 18.38 cfu∙g^−1^, 3595.0 ± 148.49 cfu∙g^−1^, and 1810.0 ± 608.11 cfu∙g^−1^, respectively. In addition, environmental *Staphylococcus* were the highest in barn 6, which had an average of 2.700 ± 0.00 cfu∙g^−1^. The present study indicates an inverse correlation between the moisture content and the bacterial counts (coliforms, r = 0.377, *p* < 0.05; *E. coli*, r = 0.448, *p* < 0.05; Bacillus, r = 0.372, *p* < 0.05). There were no significant correlations between the bacterial counts (*Streptococcal* and *Staphylococcal*) and moisture content (*p* > 0.05).

## 4. Discussion

In this descriptive study, we developed a database from 42 compost-bedded pack barns in Kentucky. This research provided an overview of a variety of features regarding the structure, environment, management, and air quality. Few studies have explored these factors. Here, we aimed to investigate the degree of heterogeneity in the conditions inside dairy production facilities, mainly for the CBP barns in an important region of the USA with a great concentration of CBP barns. Producers were asked to comment on whether they were satisfied with their barn, their perceived benefits of the CBP barn, their recommended facility changes, their recommendations to other farmers building a new facility or not having success with their current facility, and the lessons they have learned throughout the process. Of the 42 producers visited, all responded that they were satisfied with their compost-bedded pack barn, and only one stated that he was sometimes satisfied. However, a high variability in structures and environmental conditions was observed, as detailed below.

### 4.1. Barn Structure and Layout

The first CBP barn was constructed in Kentucky in 2002 and the CBP barn number had increased to 58 by the end of 2010. The type of barn construction varied, including the barn dimensions, the type of ridge, the ridge orientation, the ventilation system, etc. [1,3]. Although CBP barns have different remarkable characteristics in their layout, the bed area was the only common feature between the assessed CBP barns [1]. Most of the CBP barns (33.3%) presented a bed area between 500 and 1000 m^2^, and only 5% of the CBP barns presented a bed area > 3000 m^2^ (Figure 3). In a prior study conducted in Italy, the authors found an area of 400 m^2^, of which 340 m^2^ was bed [19]. In a study carried out by Oliveira et al. [20] in the state of Minas Gerais, Brazil, the average dimensions of the bedding area found in CBP sheds were 64.1 ± 27.1 m (length) by 17.7 ± 4.1 m (width), and the average size of the resting area was 1134.1 ± 537.6 m^2^, with an average value greater than 750.0 ± 551.1 m^2^.

The average dimensions found in the studied CBP barns were 49.1 m long by 21.9 m wide. Other studies reported similar dimensions, such as 24.4 m wide by 31.4 m long [21] and 20.2 m wide by 43.5 m long [22]. Moreover, in these studies, the number of animals in the herds was similar to that of our findings. Differently from the observed studies, Oliveira et al. [4], when evaluating CBP barns with open sides and positive pressure ventilation in Brazil, found the installation dimensions to be 60.0 m long × 27.6 m wide.

Among the limitations found in this study that limited the welfare of lactating cows, we found that almost 1/3 of the compost barns did not provide access to water for the cows directly at the CBP barn. In this case, access to water and food for the animals occurred in sheds close to the CBP barn. The recommendation is to provide a 0.60 to 1.00 m linear length of water for each group of 15 cows [23]. A cow’s water intake has a critical effect on her milk productivity and reproductive performance, since it regulates dry matter intake. A cow must have access to water when needed to satisfy this requirement. Only 19.0% met the recommendations of 0.10 to 0.15 m∙cow^−1^ [9]. The recommended feed bunk (stocking density) space per cow is 0.03 to 0.06 m∙cow^−1^ [24]. Based on this information, only 29.0% met the recommendation.

On the other hand, for 26.2% of the assessed CBP barns, the cows had access to pasture. Grazing in some regions around the world is seen as beneficial to health and welfare [25]. There is consolidated evidence that not providing cows with access to pasture puts the dairy industry at odds with social and animal welfare values [26].

In this study, it was found that more than half (52.3%) of the CBP barns had a lagoon, which was typically smaller than that present in other barns. The housing system can affect dairy cow welfare and performance and has a major influence on the ecological footprint and consumer perception of dairy farming [1]. Therefore, the use of bedding material compost from sheds as organic manure is recommended as a sustainable alternative in the reuse and treatment of waste from cattle confinement farms [27]. The CBP system is ecologically more sustainable because most animal waste is incorporated into the bed and only the waste from the feeding track, transit area, and milking area are directed to the lagoon [12]. In general, there is a 70 to 75% reduction in the volume of waste in a CBP barn when compared to a free stall [28]. In a study carried out by Bewley et al. [23] in the USA, more than half of the CBP barns had a lagoon. In Brazil, Oliveira et al. [20] conducted a similar study and identified that 70.6% of the assessed CBP barns presented a lagoon. A study on farmers’ opinions regarding cubicle and CBP systems in Austria, Germany, Italy, the Netherlands, Slovenia, and Sweden found that farmers judged the CBP system as more sustainable than the cubicle-housing system [29]. However, they believed the cost of the bedding material was a serious drawback of this system.

A barn’s orientation will determine the intensity of solar radiation falling on the walls, bed and feed alley, and roof of the building. Barns generally use an E–W orientation in Kentucky, though the recommended orientation is site-specific based upon natural wind flows. The typical prevailing winds in Kentucky come from the SW in the summer and from the NW in the winter, which would suggest the E–W ridge orientation as the most effective. A total of 50% of the assessed CBP barns in Kentucky were built in the direction considered appropriate (NE–SW) for the site. A similar result was reported by Radavelli et al. [22] in Brazil, in which 50% of the CBP barns presented an E–W orientation. Moreover, according to the same authors, this orientation prevents a high concentration of animals in specific areas of the bed, which can affect the quality of the bed and the comfort of the animals.

Winds prevailing in some geographical regions may contribute to maintaining the comfort of animals. The best use of natural ventilation in CBP barns is from the height of the sidewall between 4.0 and 5.0 m [1]. In more than half of the assessed CBP barns (66.7%), the height of the sidewall was between 2.0 and 4.0 m. The CBP barn must present these recommendations for the height of the sidewall, as these dimensions contribute to the removal of the moisture and heat created by the animals. Concerning the slope of the roof, a slope of 4:12 is recommended [24]. Barns with roof pitches higher than 4:12 or lower than 3:12 [12] can limit the natural ventilation rate per cow. It was observed that 54.8% of the CBP barns attended this recommendation.

The sidewall opening height and eave height are important for protecting the cows from the sun, rain, and wind, thus helping to keep proper ventilation and to favor animal comfort. A 3.5 m sidewall opening is recommended for barns less than 12.2 m wide, while a 4.2 m opening height is recommended for barns wider than 12.2 m [30]. The correct dimensions of the eaves is fundamental to preventing edaphoclimatic factors (rain, snow) from falling on the bed and, as a result, harm the composting process. In our study, a width of the eaves of up to 1.0 m in length predominated in 54.8% of the cases evaluated. In Brazil, Oliveira et al. [20] observed that the predominance of eave widths was from 2.0 to 3.0 m.

The ridge design affects air movement by removing warm/hot moist air that collects under the roof. The design of the ridge can help or inhibit barn ventilation depending on the wind velocity and direction relative to the ridgeline. The width of the continuous ridge opening controls the air exhausting from the barn. As the width of a barn increases, the ridge opening should also increase. The recommended ridge opening width is 0.07 m/3.05 m of barn width, with a minimum width requirement of 0.30 m for barns less than 12.20 m wide (Figure 7). The apparent opening is the ridge opening width as seen from inside the barn. The effective opening is the smallest width through which air flows to the outside of the barn at the ridge. Most barns had ridge opening restrictions that could cause inadequate natural ventilation, which can significantly affect cows, particularly under heat stress conditions.

To control thermal stress in CBP barns and promote bed drying, mechanical ventilation can be a strategy. Most of the confinements analyzed used ventilators for air circulation, and 23.8% of the CBP barns used high-volume and low-speed (HVLS) fans. HVLS fans are commonly used in CBP barns [1]. HVLS fans provide a better thermal environment to the animals, with a lower dry bulb temperature; a lower RH, temperature, and humidity index; and a reduction in the noise inside the shed [12]. However, 2.5% of the confinements had a negative pressure system. Buildings that use tunnel ventilation require more elaborate ridge openings with some type of closure system [31].

In 2015, in Brazil, some closed CBP facilities were built, equipped with negative pressure mechanical ventilation systems [2,32,33]. According to the research carried out during the summer and winter, the characteristics of the bedding indicated that the material was at undesirable levels for optimal composting, with emphasis on the high levels of litter moisture [3]. It is worth mentioning the importance that the elaboration and conduction of activities of CBP facility projects are carried out by qualified professionals, and that a careful examination is carried out on the location and climate before the implementation of the system.

### 4.2. Compost-Bedded Pack Management

In all CBP systems, special attention should be given to the bed area. The bed should allow the animals enough space to get around and be comfortable. The bed receives a daily load of waste and for this reason, the management of the same should be considered, because inadequate management can increase the moisture content of the bed, impair composting, and consequently increase the cases of dirty animals and the incidence of mastitis [34].

Among the bedding materials, materials derived from wood are considered warmed, as they have an adequate C:N relationship [1] and energy [35]. In Minnesota, the recommended bedding materials are sawdust and wood shavings [9,14]. In Kentucky, producers use green or dried shavings and sawdust [8]. In the present study, we observed that among the bed materials used, the highest percentage (45.2%) was for dry wood or sawdust, followed by green sawdust (42.9%). Wood or sawdust shavings have a high absorption capacity and can maintain the proper bed structure [1], besides being easy to obtain and inexpensive. However, the use of non-dry materials in the bed, such as green sawdust, may increase the incidence of mastitis [36] and compromise the ability to absorb water in the bed [8,9].

To form a CBP bed, it is recommended to add 0.40 to 0.50 m of material and, later, an addition of 0.10 to 0.20 m of bedding every five weeks. The CBP bed should be changed between 6 and 12 months [28]. Several producers changed the bed every 12 months and started a new bed after removal with a 0.30 m depth. Some producers left 0.15 m from the old bed to start microbial activity. Every one to five weeks, Kentucky producers added new material to the bed. Most CBP beds in Kentucky were revolved twice a day with a depth of 0.11 to 0.23 m. Bed revolving incorporates the addition of oxygen, making the composting system active [12]. Composting occurs when the bed is turned at least twice a day [15].

For bed composting to occur, the bed moisture must be between 40 and 65% [37]. In the superficial layer of the bed (0.30 m), for the composting process to take place correctly, the temperature must fluctuate between 45 and 60 °C [9]. In the present study, the superficial layer of the bed (0.20 m) was observed to have a mean moisture content among the CBP barns of 59%, ranging from 36.2 to 71.8%. Excessive moisture addition needs to be avoided. The moisture content can vary owing to the addition of fresh bedding, weather, and cow density in the sampling area [15]. Regarding the bed temperature, it was observed that in the sheds that used green sawdust, the temperature was lower. The highest temperature was in the fluffy areas of the bed. This observation is consistent with the need for oxygen for microbial activity, which promotes composting.

In this way, the bed area is one of the main parameters of attention in CBP barns [1]. The bed size is defined as the bed area per animal (m^2^∙cow^−1^). Reduced areas per animal may concentrate a greater amount of feces and urine in the bed, increasing the moisture content of the bed and making it difficult to manage it. Different bed areas per animal have been reported in the literature, such as 7.4 m^2^∙cow^−1^ [9], 8.6 m^2^∙cow^−1^ [15], 12–17 m^2^∙cow^−1^ [21], 14.6 m^2^∙cow^−1^ [22], 11–19 m^2^∙cow^−1^ [34], and 10.5 m^2^∙cow^−1^ [38]. However, if the bed area is less than 9.3 m^2^∙cow^−1^, the moisture and compaction of the bed may increase [6]. In our study, we observed an average density of 8.9 m^2^∙cow^−1^ and a maximum density of 25.8 m^2^∙cow^−1^. In addition, around 25% of the CBP barns did not present a sufficient bed area per cow. An insufficient area per animal may limit milk production [1] and, consequently, harm the comfort and animal welfare of the cows and increase competition for resting areas between the cows [23,25,39,40].

### 4.3. Environment Characteristics

The thermal environment encompasses a series of factors that characterize the microclimate inside the facilities, which interact with each other and reflect the real thermal sensation of the animals [41]. The comfort zone for dairy cows ranges from 13 to 18 °C, while the critical zone ranges from 25 to 28 °C [42]. Regarding the RH, the same authors reported that the ideal range varies between 40 and 60%. Although the air temperature varied between the measuring heights (0.05 m and 1.20 m) and between the barns, the air temperatures were lower in the CBP barns that used overshot or capped ridges, mechanical ventilation systems, and an installation in the direction of NE–SW.

Properly designed ventilation in CBP barns influences the comfort of the animals as well as the quality of the bed, assisting in its drying [43,44]. For the ventilation system to be efficient, wind speed throughout the length of the barn of around 3 m∙s^−1^ has to be provided [22]. However, considerable variation was observed in the internal and external air velocities in 38% of the analyzed barns, which can be explained by the obstacles present near the barn.

A decreased wind speed within certain limits results in an increased RH [21]. In most sheds, an increase in humidity was observed with increasing height. During the seasons of fall and winter in Kentucky, the barn orientation was not ideal for air movement, since air generally moving along the length meets a higher resistance than air moving across the building. Sufficient air exchange is needed in cold weather to remove moisture from the pack and extend the time between bedding additions. Overall, the low sidewalls of the barns promoted a high air velocity across the barns. Air movement through the barn should be sufficient to maintain the inside temperature only slightly above the outside temperature in winter, and slightly below the outside temperature in summer. On the other hand, a high airflow during the winter can reduce the air temperature indoors and the pack temperature. In those conditions, windbreaks become very important. 

### 4.4. Herd Characteristics

The average herd size (89.7 cows) in our study was similar to that found in Minnesota (73 cows) [15], and the maximum number of animals (184 cows) was similar to that found in Kentucky previously (178 cows) [28]. Most cows were Holstein (72.7%), followed by crossbred (20.5%) and Jersey (6.8%). Studies have reported the use of different breeds of dairy cattle in confinements of the CBP model [8,45,46,47,48,49]. The average production of cows (27.2 kg∙day^−1^) in our study was similar to that reported in Kentucky previously (27.3 kg∙day^−1^) [8]. The average fat (3.85%) and protein (2.35%) contents are comparable to those reported in other studies [10,45,48].

### 4.5. Producer Responses

Among the producers, 24.2% mentioned the increased comfort of cows. Others cited an increase in cow cleanliness (12.1%) and low system maintenance (9.5%), in addition to a decrease in the somatic cell count (5.2%). In Kentucky, Black et al. [8] characterized the satisfaction of the producers who used the compost barn system. They also reported that most producers were satisfied with the system. The cows were more comfortable and cleaner, and the system resulted in low maintenance. CBP barns are considered healthier to animals due to less exposure to a concrete area [23]. This system was built with the aim of better comfort and longevity of cows [9]. In Minnesota, Barberg et al. [10] observed a mean decrease of 12% in the prevalence of mastitis in animals housed in CBP barns.

When we asked the producers about their concerns with the system, most of the producers reported the size or capacity of the barn (25%). A restricted bed area results in increased compaction and moisture content [9], harms the welfare [40], and reduces animal productivity [1]. In addition, producers reported the need for a containment wall (10%), curtains (8.3%), and more fans (8.3%). The use of curtains has been adopted among producers to avoid bed watering due to rains and winds or for the protection of animals on cold days. In CBP facilities, the use of fans should provide adequate ventilation throughout the barn so that the bed can dry, gases can be removed, and heat exchanges can be favored between the animal and the environment [8]. In addition, the ventilation system should be carefully designed so that the spatial variability in the barn is reduced [21].

One of the main concerns of the producers was the supply of bedding material. Producers sought the supply of safe material (17.7%) and alternative sources (9.7%), such as kiln-dried shavings (9.7%). In addition, they cared about revolving the bed twice a day (14.5%) and maintaining adequate bed humidity (9.7%). One of the major limitations of the CBP system is obtaining quality bed material, i.e., bedding with a good C:N ratio (above 30:1) with a medium size (3 to 5 cm long and 2 to 3 mm thick), a high absorption capacity of humidity, low thermal conductivity, a low cost, and availability in the region [12].

The farmers mentioned that they exchanged experiences with other producers and that they created their system management and experience. The producer’s business-oriented mentality draws an aspect focused on technical and economic results [7]. Thus, Kentucky producers learned to use the CBP system based on its limitations, their experiences, and their mistakes.

### 4.6. Bacterial Analysis of Composts

One of the main concerns regarding CBP barns is the exposure of cows to environmental pathogens causing mastitis, due to the organic nature of the bed. Pathogens are present in animal waste and are accumulated in the organic material present in the bed. When the bed temperature exceeds 55 °C, the pathogenic microorganisms are eliminated [28]; however, when the bed temperature is <50 °C, there is a lack of a sufficient temperature to eliminate the bacteria present in the bed [38].

In Kentucky, Eckelkamp et al. [5], when analyzing eight CBP barns, identified that as the internal temperature of the bed increased, the growth of *Staphylococcal* species, *Streptococcal* species, and some species of *Bacillus* was reduced and the growth of coliform species increased. This result contradicts what was found in shed 15, in which there was no presence of coliforms and among the CBP barns analyzed, it presented the highest temperature between the beds.

The results for the bacterial species loads in barn 4 (coliforms ± 12.0 cfu∙g^−1^, *E. coli* ±18.38 cfu∙g^−1^, *Bacillus* spp. ±148.49 cfu∙g^−1^, and *Streptococcus* ±608.11 cfu∙g^−1^) are higher than the results reported in other studies [43,50,51]. More than 10 log_10_ cfu∙mL^−1^ may increase the risk of mastitis incidence in dairy cows [52]. The results for the bacterial species in shed 4 were higher than the limit (10 log_10_ cfu∙mL^−1^) for mastitis in cows. Thus, in this CBP barn, the care should be redoubled with respect to bed management and the importance of cleaning procedures for ceilings before milking should be emphasized. Therefore, bed management practices have a considerable impact on milk quality, cow hygiene, and bacterial concentrations in the bed [53]. Albino et al. [54], when assessing the relationships between bacterial populations in the bed material at the tips of the ceilings for cows housed in a CBP barn, did not observe significant correlations between the populations of *E. coli*, coliforms, *Klebsiella* spp., and *Streptococcus* spp. In addition, the authors concluded that the hygiene score in animals may not be an accurate tool to identify bacterial contamination on the tips of the ceilings or in milk. Finally, studies in CBP barns on cow hygiene have shown inconsistent results [1].

## 5. Conclusions

Through the conduct of this study, the construction layout, the bedding material, and the major interactive factors in the success of bed composting of 42 compost-bedded pack barns in Kentucky (USA) were characterized.

The compost-bedded pack (CBP) barns examined in Kentucky had a variety of building designs. The most frequent features of the barns were: an orientation of NE–SW; the presence of overshot ridges; a roof pitch of 4:12; and the presence of box fans. Air temperatures tended to be lower indoors than the barns that utilized overshot or capped ridges, forced ventilation systems, and a ridge direction of NE–SW or NW–SE. The average relative humidity in this study was approximately 65.0%. In general, the wind direction was transverse to the barn’s length direction and the air velocity was 1.0 m∙s^−1^. The bedding material most frequently used in the CBP barns was kiln-dried sawdust. The pack temperatures indicated that some of the bedding materials were not able to support microbial activity and produce heat, but the average pack moisture content was 59.0% (±9.0) on a wet basis. The bedding compost always contained bacteria, but coliforms were not present in barns that had a higher compost temperature. The coliform, E. coli, Bacillus, and Streptococcus counts were higher in the barns that showed a lower moisture content. The most frequently cited benefits of the CBP barns included increased cow comfort compared to free stalls; increased cow cleanliness; low maintenance; their ability to work well for heifers and lame, fresh, problem, and old cows; and their ability to allow a natural resting position with no free stalls.

## Figures and Tables

**Table 1:** Producer-cited benefits of 42 CBP barns.

Benefits	Percentage (%)
Improved cow comfort	24.1
Improved cow cleanliness	12.1
Low maintenance	9.5
Good for heifers, lame, fresh, problem, and old cows	8.6
Natural resting position (no stalls)	7.8
Improved feet and legs	6.9
Proximity to the parlor (compared to pasture)	6.9
Decreased SCC	5.2
Increased heat detection	5.2
Ease of manure handling	2.6
Increased dry matter intake (compared to pasture)	2.6
Increased production	2.6
Increased longevity	2.6
Fewer leg and teat injuries	1.7
Time standing on concrete minimized	1.7

SCC—somatic cell count.

**Table 2:** Recommended facility changes of CBP barns by producers.

Recommended	Percentage (%)
Increasing the size or capacity of the barn	25.0
Higher sidewalls and improved ventilation	20.0
Adding a retaining wall	10.0
Adding curtains	8.3
More fans	8.3
Larger ridge vent	8.3
No posts in a pack	6.7
Changing the number or location of waterers	6.7
Changing the location or size of the feed bunk	6.7

**Table 3:** Producer recommendations and lessons learned by CBP barn producers.

Recommendations and Lessons Learned	Percentage (%)
Secure a bedding supply	17.7
Do not use straw, wheat straw, corn fodder, bean fodder, or pine	9.7
Add bedding frequently	6.5
Tour other barns	4.8
Stir two times per day or more frequently	14.5
Keep pack maintained and moisture low	8.1
Build the barn large	6.5
Add curtains	4.8
Use kiln-dried shavings	9.7
Have a minimum of 9.3 m^2^∙cow^−1^	8.1
Designate a tractor for stirring	4.8
Do not start packing during winter	4.8

## Data Availability

The data presented in this study are available from the corresponding author upon request.
